# In Vivo Evaluation of Cerebral Hemodynamics and Tissue Morphology in Rats during Changing Fraction of Inspired Oxygen Based on Spectrocolorimetric Imaging Technique

**DOI:** 10.3390/ijms19020491

**Published:** 2018-02-06

**Authors:** Afrina Mustari, Takuya Kanie, Satoko Kawauchi, Shunichi Sato, Manabu Sato, Yasuaki Kokubo, Izumi Nishidate

**Affiliations:** 1 Graduate School of Bio-Applications & Systems Engineering, Tokyo University of Agriculture and Technology, Koganei 184-8588, Japan; s150747w@st.go.tuat.ac.jp (A.M.); s170638r@st.go.tuat.ac.jp (T.K.); 2 Division of Bioinformation and Therapeutic Systems, National Defense Medical College Research Institute, Tokorozawa 359-8513, Japan; skawauch@ndmc.ac.jp (S.K.); shunsato@ndmc.ac.jp (S.S.); 3 Graduate School of Science and Engineering, Yamagata University, Yonezawa 992-8510, Japan; msato@yz.yamagata-u.ac.jp; 4 Department of Neurosurgery, Faculty of Medicine, Yamagata University, Yamagata 990-9585, Japan; ykokubo@med.id.yamagata-u.ac.jp

**Keywords:** diffuse reflectance spectroscopy, RGB image, Monte Carlo simulation of light transport, light scattering, cerebral hemodynamics, hemoglobin oxygen saturation, brain tissue viability, anoxic depolarization

## Abstract

During surgical treatment for cerebrovascular diseases, cortical hemodynamics are often controlled by bypass graft surgery, temporary occlusion of arteries, and surgical removal of veins. Since the brain is vulnerable to hypoxemia and ischemia, interruption of cerebral blood flow reduces the oxygen supply to tissues and induces irreversible damage to cells and tissues. Monitoring of cerebral hemodynamics and alteration of cellular structure during neurosurgery is thus crucial. Sequential recordings of red-green-blue (RGB) images of in vivo exposed rat brains were made during hyperoxia, normoxia, hypoxia, and anoxia. Monte Carlo simulation of light transport in brain tissue was used to specify relationships among RGB-values and oxygenated hemoglobin concentration (*C*_HbO_), deoxygenated hemoglobin concentration (*C*_HbR_), total hemoglobin concentration (*C*_Hb__T_), hemoglobin oxygen saturation (*StO*_2_), and scattering power *b*. Temporal courses of *C*_HbO_, *C*_HbR_, *C*_HbT_, and *StO*_2_ indicated physiological responses to reduced oxygen delivery to cerebral tissue. A rapid decrease in light scattering power *b* was observed after respiratory arrest, similar to the negative deflection of the extracellular direct current (DC) potential in so-called anoxic depolarization. These results suggest the potential of this method for evaluating pathophysiological conditions and loss of tissue viability.

## 1. Introduction

Cerebrovascular diseases (CVDs) affect the blood vessels and blood circulation in brain tissues. Common CVDs include ischemic stroke, transient ischemic attack, and subarachnoid hemorrhage. Stroke was the second most common cause of death worldwide in 2015. Along with ischemic heart disease, CVDs have remained a leading cause of death globally for the last 15 years [1]. CVDs can damage and deform cerebral arteries delivering oxygen and nutrients to the brain tissues. Neuronal cells demand a continuous supply of oxygen and glucose, which are delivered via the blood circulation. Impaired cerebral blood circulation can cause irreversible cell injury or cell death as a consequence of neuronal energy failure [2]. Various treatment strategies for CVDs, including medication, lifestyle changes and/or surgery, are available depending on the specific underlying cause. In surgical treatments for CVDs, cortical hemodynamics are often controlled by bypass graft surgery, temporary occlusion of arteries, or surgical removal of veins. Since the brain is vulnerable to hypoxemia and ischemia, interruption of cerebral blood flow reduces the oxygen supply to tissues and can induce irreversible damage to cells and tissues. Monitoring of cerebral hemodynamics and alterations to cellular structure during neurosurgery is thus extremely important.

Cerebral hemodynamics, including cerebral blood flow, cerebral blood volume, oxygen saturation of hemoglobin in red blood cells (RBCs), affect the light-absorbing properties of brain tissues, whereas tissue morphology can be associated with light-scattering properties. The optical properties of cerebral tissues have therefore been used to perform spatiotemporal mapping of neuronal activity and tissue viability in the brain as intrinsic optical signals (IOSs). IOSs in the brain are thought to be caused primarily by the following factors: hemodynamic-related changes in absorption properties, changes in light scattering induced by cellular deformations caused by water movement between the intra- and extracellular compartments, such as cell swelling or shrinkage, and changes in absorption due to redox states of cytochromes in the mitochondria [3].

The effective scattering coefficient spectrum *μ_s_*′ (*λ*) of biological tissues is considered to be the product of the combination of *μ_s_*′ (*λ*) for the tissue ultrastructure of different sizes [4]. The spectra of *μ_s_*′ (*λ*) in biological soft tissues follow a power law function [5,6] that can be approximated in the following form:

(1) $${\mu_{s}}^{\prime }=a\lambda^{-b}$$

where *a* is the scattering amplitude and *b* is the scattering power, which are related to geometrical properties such as scatterer density [7] and size [8], respectively. A decrease or increase in scattering power *b* reportedly produces an increase or decrease in scatterer size, respectively [8]. Quantification of *b* is thus useful to evaluate morphological changes such as cell swelling or shrinkage in cerebral tissue. 

Light in the visible to near-infrared spectrum is susceptible to the absorption and scattering properties of biological tissues. Estimation of the scattering and absorption properties of in vitro tissue slice samples from measured diffuse reflectance and transmittance of tissue slices [9] has been reported based on various approaches for solving light transport in turbid media, such as the diffusion approximation to the radiative transport equation [10], the adding-doubling method [11], and the Monte Carlo simulation (MCS) [12]. Numerous optical methods have been reported for determining scattering and absorption properties in living tissues, including time-domain spectroscopy [13], frequency-domain spectroscopy [14], and spatially resolved spectroscopic techniques with continuous wave (CW) light [15,16,17,18,19,20,21]. 

Diffuse reflectance spectroscopy (DRS) can be simply achieved with a light source emitting CW light, simple optical components, and a spectrophotometer. DRS is one of the most promising methods for determining the optical properties of brain tissue in vivo. Several methods using a lookup table method based on the MCS of light transport have been proposed for estimating the absorption and scattering properties of biological tissues [22,23,24,25]. Multispectral imaging systems based on DRS have been utilized to visualize cortical hemodynamics based on changes in the absorption properties of chromophores in brain tissues [26,27,28,29,30,31]. An acousto-optical tunable filter [32] and the combination of a lenslet array with narrowband filters [33] have been introduced as spectroscopic elements to achieve rapid multispectral imaging. On the other hand, estimation of multispectral images from a red-green-blue (RGB) image acquired using a digital color camera has begun to attract attention as a method for rapid yet cost-effective imaging. A multispectral imaging technique based on the Wiener estimation method has been applied to visualize the cortical hemodynamics and light-scattering properties of in vivo rat brain [34]. However, this method is time-consuming for analyzing spectral data for each pixel of a multispectral image cube to reconstruct a set of images of oxygenated hemoglobin, deoxygenated hemoglobin, and scattering properties.

The present study investigated a simple and rapid imaging method for oxygenated hemoglobin concentration (*C*_HbO_), deoxygenated hemoglobin concentration (*C*_HbR_), total hemoglobin concentration (*C*_HbT_), hemoglobin oxygen saturation (*StO*_2_), and scattering power *b* of in vivo exposed brain tissues based on DRS using a digital RGB camera. In this method, MCS of light transport in homogeneous tissue was introduced to specify relationships among RGB values and *C*_HbO_, *C*_HbR_, and *b*. *C*_HbT_ and *StO*_2_ were also calculated from estimated values of *C*_HbO_ and *C*_HbR_. To confirm the feasibility of a method by which to evaluate cerebral hemodynamics and morphological changes in brain tissues, we performed in vivo experiments using exposed rat brain while changing the fraction of inspired oxygen (FiO_2_) to include conditions of normoxia, hypoxia, and anoxia.

## 2. Results and Discussion

**Figure 1:**
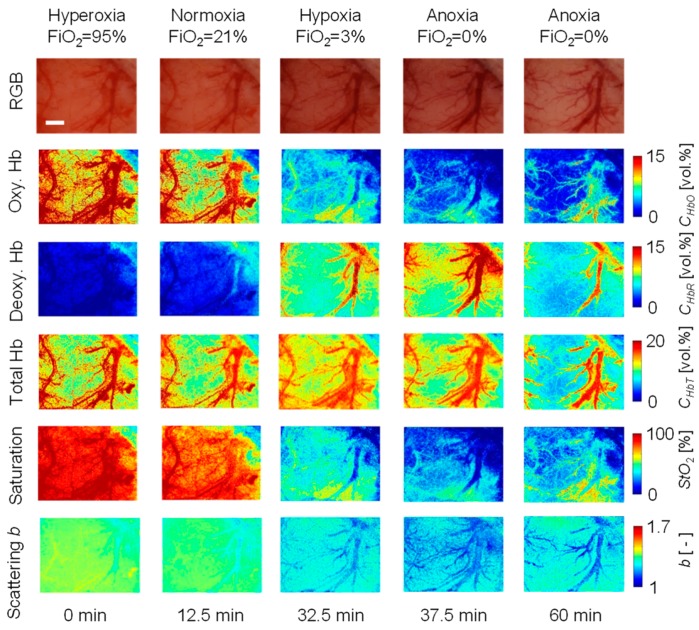
Typical images obtained during hyperoxia, normoxia, hypoxia, and anoxia for the RGB color image, *C*_HbO_, *C*_HbR_, *C*_HbT_, *StO*_2_, and *b*. A scale bar (white line in the RGB image for 0 min) indicates 1.0 mm.

**Figure 2:**
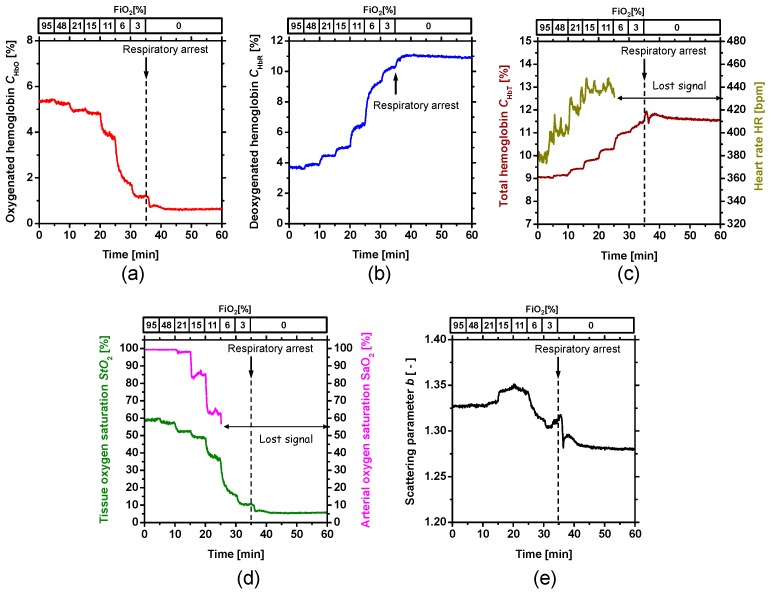
Typical time courses of (**a**) oxygenated hemoglobin *C*_HbO_, (**b**) deoxygenated hemoglobin *C*_HbR_, (**c**) total hemoglobin *C*_HbT_ and heart rate HR, (**d**) hemoglobin oxygen saturation *StO*_2_ and (**e**) arterial oxygen saturation *SaO*_2_, and scattering power *b* averaged over the area for the region of interest (ROI) on the parenchymal region.

Figure 1 shows typical in vivo resultant images for *C*_HbO_, *C*_HbR_, *C*_HbT_, *StO*_2_, and *b* during changes in FiO_2_. Figure 2 shows the time courses of (a) *C*_HbO_, (b) *C*_HbR_, (c) *C*_HbT_, (d) *StO*_2_, and (e) *b* averaged over the area for the ROI in the parenchyma during changes in FiO_2_. Time courses of *SaO*_2_ and HR were also compared with *StO*_2_ and *C*_HbT_, respectively, in Figure 2. Values of *C*_HbO_ and *C*_HbR_ decreased and increased, gradually, as FiO_2_ decreased, which caused decreases in *StO*_2_. On the other hand, the value of *SaO*_2_ dropped dramatically when FiO_2_ was below 21%, indicating the onset of hypoxemia due to hypoxia. The value of *C*_HbT_ gradually increased according to reductions in FiO_2_. The value of HR gradually increased after the onset of hypoxia and reached a maximum amplitude approximately one minute after respiratory arrest (RA), implying an increase in blood flow compensating for hypoxia. Immediately following RA, values of both *C*_HbO_ and *C*_HbT_ dropped sharply. Time courses of *C*_HbO_, *C*_HbR_, *C*_HbT_, and *StO*_2_ while changing FiO_2_ were consistent with well-known physiological responses to changes in FiO_2_. As shown in Figure 2e, the value of *b* began to increase after the onset of anoxia, then decreased rapidly after RA. The change in *b* is independent of the hemodynamic parameters of *C*_HbO_, *C*_HbR_, *C*_HbT_, and *StO*_2_. 

**Figure 3:**
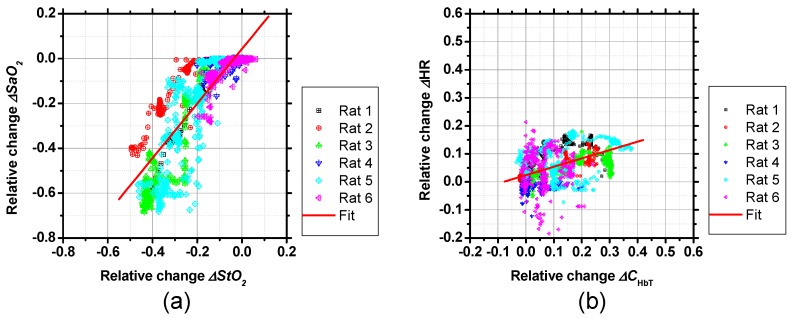
Scatter plots of relative changes in (**a**) *SaO*_2_ vs. *StO*_2_ and (**b**) *C*_HbT_ vs. HR, obtained from the six rats.

Figure 3 shows scatter plots of relative changes in (a) *SaO*_2_ vs. *StO*_2_ and (b) *C*_HbT_ vs. HR, obtained from the six rats. As shown in Figure 3a, values of *StO*_2_ correlated well with values of *SaO*_2_. The correlation coefficient between *SaO*_2_ and *StO*_2_ was *r* = 0.85 (*p* < 0.0001). Figure 3b shows that *C*_HbT_ tends to increase with increasing HR. The correlation coefficient between *C*_HbT_ and HR was *r* = 0.5 (*p* < 0.0001), indicating that *C*_HbT_ is moderately correlated with HR.

**Figure 4:**
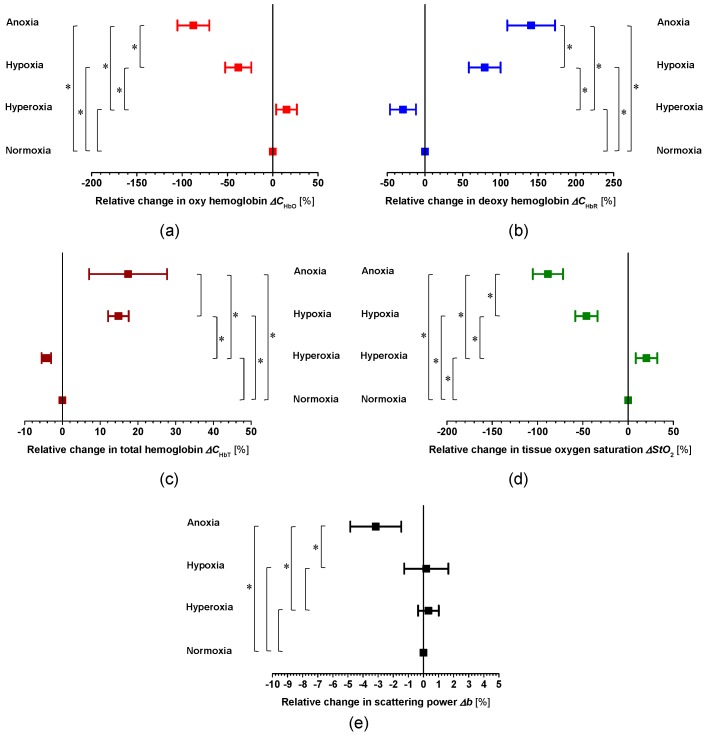
Relative change in (**a**) oxygenated hemoglobin *∆C*_HbO_, (**b**) deoxygenated hemoglobin *∆C*_Hb__R_, (**c**) total hemoglobin *∆C*_Hb__T_, (**d**) hemoglobin oxygen saturation *∆StO*_2_, and (**e**) scattering power *b* averaged over the ROIs for all six samples under conditions of hyperoxia, hypoxia, and anoxia. Error bars show standard deviations (*n* = 6). * *p* < 0.01.

Figure 4 shows time averages over the period of hyperoxia (FiO_2_ = 95%), normoxia (FiO_2_ = 21%), hypoxia (FiO_2_ = 10%), anoxia (FiO_2_ = 0%) after RA for (a) *C*_HbO_, (b) *C*_HbR_, (c) *C*_HbT_, (d) *StO*_2_, and (e) *b* averaged over the ROIs for all six samples. The trends in *C*_HbO_, *C*_HbR_, *C*_HbT_, *StO*_2_, and *b* shown in Figure 1 and Figure 2 were apparent in all six samples. The average value of *StO*_2_ during normoxia over all six samples was 54.03 ± 7.49%, close to the value of around 60% reported in the literature [6,35]. As shown in Figure 2, for example, the value of *C*_HbO_ under hyperoxia (FiO_2_ = 95%) was larger than that under normoxia (FiO_2_ = 21%). On the other hand, the value of *C*_HbO_ under hypoxia (FiO_2_ = 10%) was smaller than that under normoxia (FiO_2_ = 21%). That is why the relative changes in *C*_HbO_, *C*_HbR_, and *StO*_2_ for hyperoxia showed the opposite direction than those for the other conditions, such as hypoxia and anoxia. Relative changes in *C*_HbO_, *C*_HbR_, *C*_HbT_, *StO*_2_, and *b* were calculated based on time averages over the period under each condition. Time courses of *C*_HbO_, *C*_HbR_, *C*_HbT_, *StO*_2_, and *b* fluctuate temporally in each period, even if the value of FiO_2_ remains constant, which can be a potential source of errors in calculation not only for baseline values of *C*_HbO_, *C*_HbR_, *C*_HbT_, *StO*_2_, and *b*, but also for relative changes.

**Figure 5:**
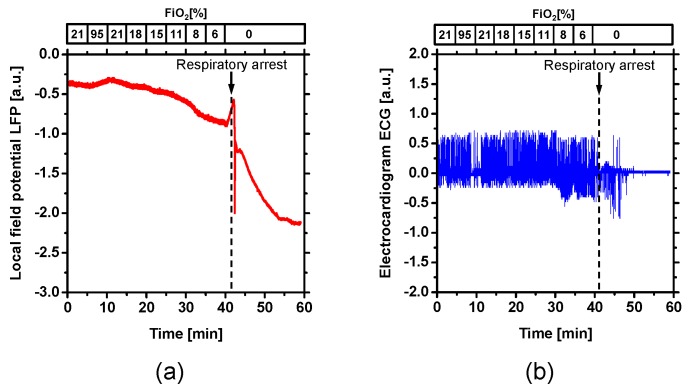
Typical time courses of (**a**) electrical local field potential (LFP) and (**b**) electrocardiogram (ECG) during changes in FiO_2_.

Figure 5 shows the typical time courses of electrocardiogram (ECG) and LFP during changes in FiO_2_. The ECG signal was irregularly disrupted immediately after respiratory arrest, whereas LFP rapidly dropped after respiratory arrest. This decrease in LFP is called anoxic depolarization (AD), and is related to brain tissue damage [36,37].

Of note is the fact that after the onset of anoxia, the time course of *b* showed a similar temporal change to that of LFP. Moreover, the average period between RA and the onset of AD was close to that between RA and the onset of the rapid decrease in *b*. The change in light scattering after respiratory arrest shown in Figure 2e therefore implies morphological changes in brain tissue induced by AD. Inhibition of mitochondrial respiration occurred under anoxic conditions due to the rapid drop in O_2_ tension, resulting in adenosine triphosphate (ATP) depletion. Decreased ATP synthesis due to the inhibition of mitochondrial respiration leads to failure of the Na^+^/K^+^ ATPase pump [36]. Under such circumstances, extracellular Na^+^, Cl^−^, and Ca_2_^+^ rush in, with water following osmotically, causing cell swelling [38,39]. The interstitial space is also reportedly halved at the time of rapid decreases in LFP due to AD [40]. The decrease in *b* after the onset of anoxia as shown in Figure 2e and Figure 4e is thus most likely caused by cell swelling and decreases in the interstitial space due to failure of the Na^+^/K^+^ ATPase pump. These results indicate the potential of the present method to evaluate electrical depolarization due to losses of tissue viability as well as cerebral hemodynamics. 

The present method is based on the MCS model, which assumes uniformly distributed scattering and absorption properties. Moreover, this approach relies on integration of all diffusing reflection information along the depth direction. As a result, the method lacks depth resolution. The depth and diameter of blood vessels usually differ among samples and may change with the age of the rat. Correct estimation is essential to precise estimation of the concentrations of oxygenated and deoxygenated hemoglobin for blood vessel regions. In the present study, we assumed the scattering spectrum of in vivo brain tissue using a power law function. This assumption may be applicable to the parenchymal region with a low volume concentration of blood. However, the diffuse reflectance spectrum from the blood vessel region with a higher volume concentration of blood can be strongly influenced by the scattering properties of red blood cells (RBCs). RBCs reportedly behave as strong scatterers of light, and the reduced scattering coefficient spectrum has a similar wavelength dependence on the absorption spectrum of hemoglobin [41]. Using the empirical formulas determined by the MCS with a heterogeneous structure model in which the scattering spectrum of RBCs may provide more reliable estimations of scattering power *b* for blood vessel regions. 

In this MCS model, the absorption coefficients of whole blood with a hematocrit of 44% were assumed to be equivalent to those of the cerebral cortex for cases in which total hemoglobin concentration was 100%. However, plasma skimming leads to a wide variability in hematocrit in the actual cortical microcirculation. This method will thus underestimate the total hemoglobin concentration when hematocrit is decreased under conditions of constant total hemoglobin concentration. Evaluating both total hemoglobin concentration and hematocrit value simultaneously is thus difficult. This study focused on evaluating changes in hemoglobin concentration and hemoglobin oxygen saturation qualitatively as a first step. We also measured arterial oxygen saturation and HR to confirm whether the results of hemoglobin concentration and hemoglobin oxygen saturation obtained from the proposed method are not inconsistent with systemic hemodynamics. We also believe that experiments with a tissue-mimicking phantom are needed to quantitatively validate the results. We have developed an agarose-based phantom that mimics the optical properties of biological tissues [31]. In the preliminary experiments, we attempted to change hemoglobin oxygen saturation in phantoms using Na_2_S_2_O_4_ solution. However, the measured diffuse reflectance spectra and RGB values tended to exhibit unexpected fluctuations and maintaining a stable condition of the deoxygenated spectra at the specific hemoglobin oxygen saturation was difficult. Validation of the method with a phantom closer to realistic conditions of brain tissue is warranted in future work.

## 3. Materials and Methods 

### 3.1. Relationship between Red-Green-Blue (RGB) Values and Optical Properties of Cerebral Tissue

RGB values of a pixel from an image of exposed cerebral cortex acquired by a digital camera can be expressed as

(2) $$R_{m}=\int E(\lambda )S_{R}(\lambda )O_{m}(\lambda )d\lambda$$

(3) $$G_{m}=\int E(\lambda )S_{G}(\lambda )O_{m}(\lambda )d\lambda$$

and

(4) $$B_{m}=\int E(\lambda )S_{B}(\lambda )O_{m}(\lambda )d\lambda$$

where *λ*, *E* (*λ*), and *O*_m_ (*λ*) are the wavelength, spectral distribution of illuminant, and measured diffuse reflectance spectrum of the cerebral cortex, respectively. *S*_R_ (*λ*), *S*_G_ (*λ*), and *S*_B_ (*λ*) are the spectral sensitivities of the red, green, and blue channels, respectively. Integrals are executed over the visible spectrum (400 to 700 nm). Assuming that the cerebral cortex contains oxygenated hemoglobin, deoxygenated hemoglobin, and biological light scatterers, the diffuse reflectance obtained from the exposed cortical surface *O*_m_ can be expressed as

(5) $$O_{m}=\frac{I}{I_{0}}=\int_{0}^{\infty }p\left({\mu_{s}}^{\prime },\mu_{a},l\right)\exp\left(-\left(\mu_{a,\mathrm{HbO}}+\mu_{a,\mathrm{HbR}}+{\mu_{s}}^{\prime }\right)l\right)dl$$

where, *I*_0_ and *I* are the incident and diffusely reflected light intensities, respectively, *p* (*μ_s_*′, *μ_a_*, *l*) is the path length probability function that depends on the optical properties as well as the geometrical condition of the measurements, and *μ_s_*′, *μ_a_*, and *l* are the effective scattering coefficient, absorption coefficient, and photon path length, respectively. Subscripts HbO and HbR denote oxygenated hemoglobin and deoxygenated hemoglobin, respectively. The absorption coefficient of chromophore is defined as the product of concentration *C* and extinction coefficient *ε* as

(6) $$\mu_{a}=C\varepsilon$$

Therefore, the values of *R*_m_*, G*_m_*,* and *B*_m_, are expressed as the functions of *C*_HbO_, *C*_HbR_, and *b*, based on Equations (1)–(6).

### 3.2. Estimation of Hemoglobin Concentrations and Scattering Parameter Based on RGB Images

**Figure 6:**
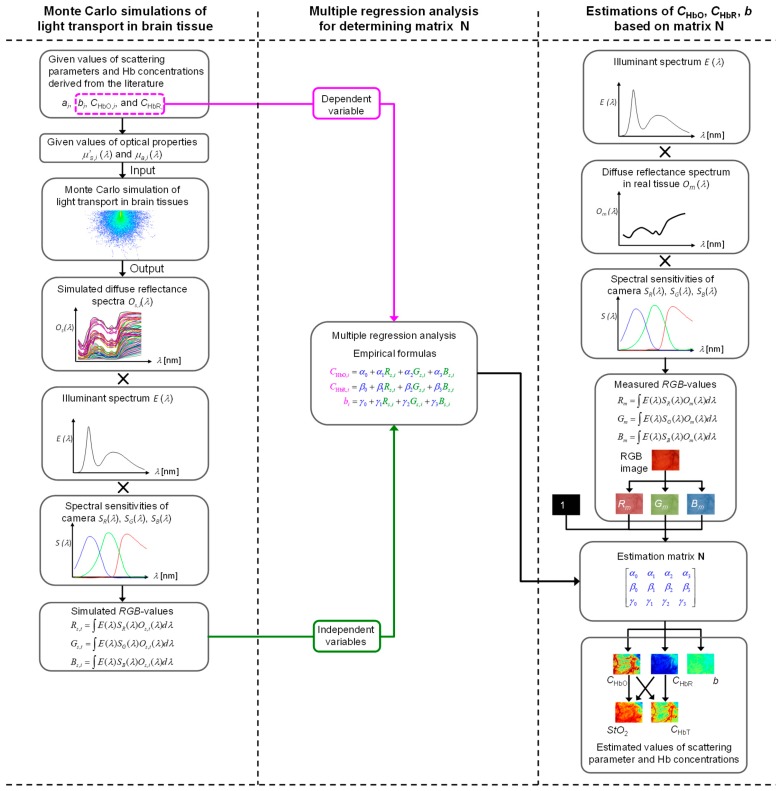
Flow diagram of the process for estimating the concentration of oxygenated hemoglobin *C*_HbO_, the concentration of deoxygenated hemoglobin *C*_HbR_, the concentration of total hemoglobin *C*_HbT_, hemoglobin oxygen saturation *StO*_2_, and scattering power *b*.

Figure 6 shows the flow of estimation by this method. In this method, values of *C*_HbO_, *C*_HbR_, and *b* are estimated from the values of *R*_m_, *G*_m_, and *B*_m_ by multiplication with matrix **N** as for each pixel of the image. Specifying the elements of matrix **N** based on Equations (1)–(6) is difficult because *p* (*μ_s_*′, *μ_a_*, *l*) and *l* are usually unavailable. We therefore simulated 450 diffuse reflectance spectra *O*_m_ (*λ*) in the wavelength range of 400 to 700 nm at 10-nm intervals with MCS of light transport [7] in brain tissue. The simulation model used here consisted of a single layer of brain tissue in which absorption and scattering properties were uniformly distributed. The MCS used in this study is a method for modeling light propagation in biological tissues and is able to solve the radiative transport equation stochastically. The simulation is based on the random walks made by photons as they propagate through tissue, which are chosen by statistically sampling probability distributions for step size *∆l* and angular deflection per scattering event. The value of *∆l* represents the distance between two successive interactions, or scattering in the tissue. The MCS is based on macroscopic optical properties such as the absorption coefficient spectrum *μ_a_* (*λ*) and the effective scattering coefficient spectrum *μ_s_*′ (*λ*), which are assumed to extend uniformly over tissue volume. Once a photon with initial intensity *I*_0_ is launched in the tissue, it is moved to a step size where it may be scattered, absorbed, internally reflected, or transmitted out of the tissue.

(7) $$\left[\begin{array}{c} C_{\mathrm{HbO}}\\ C_{\mathrm{HbR}}\\ b \end{array}\right]=N\left[\begin{array}{c} 1\\ R_{m}\\ G_{m}\\ B_{m} \end{array}\right]$$

Step size *∆l* for each photon step follows Beer’s law. Assuming isotropic scattering, the probability is proportional to exp{−(*μ_a_* + *μ_s_*′)*∆l*}. A function of a random variable (*ξ*_1_) uniformly distributed between 0 and 1, which yields a random variable with this distribution is

(8) $$\Delta l=\frac{-\ln\xi_{1}}{{\mu_{s}}^{\prime }+\mu_{a}}$$

Once step size *∆l* is determined, the photon is moved in the tissue and the position of the photon is updated. When the photon has reached an interaction site, a fraction of the photon intensity (energy of photon) *∆I*_0_ is absorbed by the interaction site, calculated as,

(9) $$\Delta I_{0}=\left(\frac{\mu_{a}}{{\mu_{s}}^{\prime }+\mu_{a}}\right)\cdot I_{0}$$

Therefore, photon intensity after the *k*-th scattering event is expressed as,

(10) $$I_{k}={\left(\frac{{\mu_{s}}^{\prime }}{{\mu_{s}}^{\prime }+\mu_{a}}\right)}^{k-1}\cdot I_{0}$$

Once the photon has reached an interaction site and its intensity has decreased, the photon with new intensity is ready to be scattered. There will be a deflection angle, *θ* (0 ≤ *θ* *< π*), and an azimuthal angle, *ψ* (0 ≤ *ψ* *<* *2π*), to be sampled statistically. The probability distribution for the cosine of the deflection angle, cos*θ*, is described by the scattering phase function. Assuming isotropic scattering, the cos*θ* is expressed as a function of the random number, *ξ*_2_:

(11) $$\cos\theta =2\xi_{2}-1$$

The azimuthal angle, *ψ*, is uniformly distributed over the interval 0 to 2*π*, is sampled as a function of the random number, *ξ*_3_:

(12) $$\psi =2\pi \xi_{3}$$

The photon is repeatedly moved until it either escapes from or is absorbed completely by the tissue. The intensity of the escaped photon from the surface of the tissue is recorded as *I*. The ratio of *I* and *I*_0_ is calculated as diffuse reflectance *O*. The MCS relies on calculating the propagation of a large number of photons by the computer, because it is statistical in nature. After propagating many photons, net distribution of all photon paths yields an accurate approximation to reality. In this study, 5,000,000 photons were launched in a single simulation of diffuse reflectance at each wavelength.

The absorption coefficient spectrum *μ_a_* (*λ*) of tissue is related to light absorption by both oxygenated hemoglobin and deoxygenated hemoglobin. Thus, hemodynamics regarding this MCS model show changes in hemoglobin concentration per unit volume and hemoglobin oxygen saturation. Hemodynamic and tissue oxygenation parameters such as blood pressure, RBC flow velocity, oxygen tension, and tissue oxygen tension in the simulation as reported by Gould et al. [42] were not considered in this MCS. Values of *μ_a_* (*λ*) derived from the concentrations of *C*_HbO_ and *C*_HbR_, and known extinction coefficient spectra of *ε*_HbO_ and *ε*_HbR_ published in the literature [43] were used as inputs of absorption properties to the simulations of the diffuse reflectance spectrum *O*_s_ (*λ*). The effective scattering coefficient spectrum *μ_s_*′ (*λ*) of tissue is associated with light scattering by tissue. The values of *μ_s_*′ (*λ*) deduced by the combinations of *a* and *b* were used as inputs of scattering properties to the simulations of the diffuse reflectance spectrum *O*_s_ (*λ*).

Scattering parameters *a* and *b* were derived from the measured scattering spectrum of biological tissue published in the literature [4]. In the MCS model, the typical values of *a* and *b* were obtained by applying Equation (1) to the measured scattering spectrum. Five different values of 6.0 × 10^4^, 9.0 × 10^4^, 12 × 10^4^, 15 × 10^4^, and 18 × 10^4^ cm^−1^ were calculated by multiplying the typical value [4] of *a* by 0.5, 0.75, 1.0, 1.25, and 1.5, respectively, whereas the five values of 1.24, 1.31, 1.38, 1.45, and 1.52 were calculated by multiplying the typical value [4] of *b* by 0.5, 0.75, 1.0, 1.25, and 1.5, respectively. 

Absorption coefficient spectra of oxygenated hemoglobin *μ_a_*_,HbO_ (*λ*) and deoxygenated hemoglobin *μ_a_*_,HbR_ (*λ*) were obtained as measured values published in the literature [43]. The total hemoglobin concentration (*C*_HbT_) can be simply calculated as

(13) $$C_{\mathrm{HbT}}=C_{\mathrm{HbO}}+C_{\mathrm{HbR}}$$

The absorption coefficient spectrum of total hemoglobin *μ_a_*_,HbT_ (*λ*) was also calculated as the sum of the absorption coefficients of oxygenated hemoglobin *μ_a_*_,HbO_ (*λ*) and deoxygenated hemoglobin *μ_a_*_,HbR_ (*λ*). Absorption coefficients of 150 g/L hemoglobin [43], corresponding to a 44% hematocrit value, were assumed equivalent to that of the cerebral cortex for the case in which *C*_HbT_ = 100%. Three different conditions of *μ_a_*_,HbT_ (*λ*) for *C*_HbT_ = 5%, 10%, and 20% were considered by multiplying the value of *μ_a_*_,HbT_ (*λ*) for *C*_HbT_ = 100% by 0.05, 0.1, and 0.2, respectively.

Cerebral hemoglobin oxygen saturation (*StO*_2_) was determined as

(14) $$StO_{2}=\frac{C_{\mathrm{HbO}}}{C_{\mathrm{HbT}}}\times 100$$

The six values of 0%, 20%, 40%, 60%, 80%, and 100% were chosen as *StO*_2_% for the simulation. In total, 450 diffuse reflectance spectra *O*_s_ (*λ*) over a wavelength range from 400 to 700 nm at intervals of 10 nm were numerically calculated. Simulated values of *R*_s_, *G*_s_, and *B*_s_ were calculated from the simulated diffuse reflectance spectrum *O*_s_ (*λ*) based on Equations (2)–(4). In this calculation, spectral distribution of the illuminant, *E*(*λ*) and spectral sensitivities of red, green, blue channels, *S*_R_ (*λ*), *S*_G_ (*λ*), and *S*_B_ (*λ*) of the camera used for the actual imaging system were considered. RGB values were then determined based on the simulated *O* (*λ*). The above procedures were carried out for 450 combinations of *C*_HbO_, *C*_HbR_, *a*, and *b* to prepare the data sets of chromophore concentrations and RGB values. To statistically specify the relationship between RGB values and *C*_HbO_, *C*_HbR_, and *b*, we performed multiple regression analysis. In the multiple regression analysis, the set of given values of *C*_HbO__,*i*_, *C*_HbR__,*i*_, or *b_i_* (*i* = 1, 2, …, 450) in the MCS model were used as the dependent variable whereas the simulated values of *R*_s,*i*_, *G*_s,*i*_, and *B*_s,*i*_, (*i* = 1, 2, ..., 450) were used as the independent variables. Three multiple regression equations, as empirical formulae for *C*_HbO_, *C*_HbR_, and *b*, were determined using the analysis:

(15) $$C_{\mathrm{HbO},\;i}=\alpha_{0}+\alpha_{1}R_{s,\;i}+\alpha_{2}G_{s,\;i}+\alpha_{3}B_{s,\;i}$$

(16) $$C_{\mathrm{HbR},\;i}=\beta_{0}+\beta_{1}R_{s,\;i}+\beta_{2}G_{s,\;i}+\beta_{3}B_{s,\;i}$$

(17) $$b_{i}=\gamma_{0}+\gamma_{1}R_{s,\;i}+\gamma_{2}G_{s,\;i}+\gamma_{3}B_{s,\;i}$$

Coefficients *α_i_*, *β_i_*, and *γ_i_* (*i* = 0, 1, 2, 3) reflect the contributions of RGB values to *C*_HbO_, *C*_HbR_, and *b*, respectively, and were used as the elements of a 4 × 3 matrix **N**, as

(18) $$N=\left[\begin{array}{c} \alpha_{0}\\ \beta_{0}\\ \gamma_{0} \end{array}\begin{array}{c} \alpha_{1}\\ \beta_{1}\\ \gamma_{1} \end{array}\begin{array}{c} \alpha_{2}\\ \beta_{2}\\ \gamma_{2} \end{array}\begin{array}{c} \alpha_{3}\\ \beta_{3}\\ \gamma_{3} \end{array}\right]$$

Transformation with **N** from RGB values to chromophore concentrations and scattering power *b* is thus expressed as

(19) $$\left[\begin{array}{c} C_{\mathrm{HbO}}\\ C_{\mathrm{HbR}}\\ b \end{array}\right]=\left[\begin{array}{c} \alpha_{0}\\ \beta_{0}\\ \gamma_{0} \end{array}\begin{array}{c} \alpha_{1}\\ \beta_{1}\\ \gamma_{1} \end{array}\begin{array}{c} \alpha_{2}\\ \beta_{2}\\ \gamma_{2} \end{array}\begin{array}{c} \alpha_{3}\\ \beta_{3}\\ \gamma_{3} \end{array}\right]\left[\begin{array}{c} 1\\ R\\ G\\ B \end{array}\right]$$

Camera responses of red, green, and blue channels vary with changes in diffuse reflectance at a shorter wavelength, middle wavelength, and longer wavelength, respectively. Diffuse reflectance spectrum is influenced by the absorption and scattering spectra. The absorption spectrum is related to the values of *C*_HbO_ and *C*_HbR_, whereas the scattering spectrum is changed by the value of *b*, which is associated with tissue morphology. The physical meaning of matrix **N** is thus the degree of connection between the RGB values and *C*_HbO_, *C*_HbR_, and *b*, respectively. Once we determine matrix **N**, images of *C*_HbO_, *C*_HbR_ and *b* are reconstructed without the MCS. The images of total hemoglobin concentration and hemoglobin oxygen saturation can be obtained based on Equations (13) and (14), respectively. 

### 3.3. Imaging System

**Figure 7:**
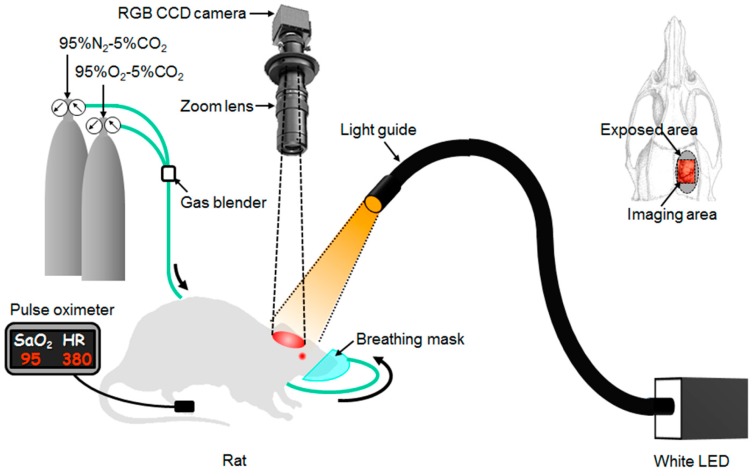
Schematic diagram of the experimental system and representation of the rat skull with a color photograph of the exposed rat brain. Red-green-blue (RGB); light-emitted diode (LED); heart rate (HR).

Figure 7 shows a schematic diagram of the experimental system used in the present study. A white-light-emitting diode (LA-HDF5010; Hayashi Watch Works, Tokyo, Japan) illuminated the surface of the exposed cortex via a light guide. The angle of illumination was approximately 45° with respect to the cortical surface, to exclude specular reflection. Diffusely reflected light from the brain was captured with a 24-bit RGB charge coupled device camera (DFK-31BF03.H; Imaging Source LLC, Charlotte, NC, USA) with a zoom lens to acquire an RGB image. A standard white diffuser with 99% reflectance (SRS-99-020; Labsphere, North Sutton, NH, USA) was used as a reference material to calibrate the white balance of the camera. RGB images were acquired with a temporal resolution of eight frames per second (fps) and stored in a personal computer. An RGB image was acquired at an exposure time of 65 ms, for a temporal resolution of 15 fps. The field of view of the system was 5.45 × 4.09 mm^2^ with 1024 × 768 pixels. Lateral resolution of the images was estimated as 5.3 μm.

### 3.4. Animal Experiments

Animal care and experimental procedures were approved by the Animal Research Committee of Tokyo University of Agriculture and Technology (Approval number 22-28, 07 May 2010 and Approval number 23-67, 21 October 2011). Six adult male Wistar rats (body weight, 202 to 402 g) were anesthetized with 5.0% isoflurane and maintained with 2.0% isoflurane. The rat’s head was placed in a stereotaxic frame. A longitudinal incision of approximately 20 mm in length was made along the midline of the head. The skull bone overlying the parietal cortex was removed with a high-speed drill to form an ellipsoidal cranial window (major axis, 8.0 mm; minor axis, 6.0 mm). The cranial window was bathed with normal saline. Respiratory condition was changed by regulating the FiO_2_ with the mixture of 95%O_2_-5%CO_2_ gas and 95%N_2_-5%CO_2_ gas and monitored by an oxygen gas sensor. The sequential RGB images measured were used to estimate images of *C*_HbO_, *C*_HbR_, *C*_HbT_, *StO*_2_ and *b* according to the process described in Figure 6. Simultaneously with optical imaging for cerebral tissue, arterial oxygen saturation (SaO_2_) and heart rate (HR) were measured by a pulse oximeter (MOUSEOX Pulse Oximeter; Star Life Science, Oakmont, PA) as systemic physiological parameters. During experiments, the presence of respiration of the rat was monitored by observing periodical movements of the lateral region of the abdomen to check for respiratory arrest.

Apart from optical imaging for cerebral tissue, measurements of electrophysiological signals were performed with five adult male Wistar rats (body weight, 294 to 594 g) while changing FiO_2_. Extracellular local field potential (LFP) was recorded by a single Ag/AgCl electrode with a spherical tip (tip diameter: 1 mm). The recording electrode was placed on the cortex while taking care to avoid large blood vessels. An Ag/AgCl reference electrode (RC5; World Precision Instruments, Sarasota, FL, USA) was attached in the neck muscle. The raw LFP signal was amplified at 1 to 100 Hz using a differential amplifier (DAM50; World Precision Instruments) under the DC mode and was digitized at 5 Hz using a data logger (midi LOGGER GL 900; GRAPHTEC, Yokohama, Japan) connected to a personal computer running GL900 application software. Electrocardiograms were measured using a differential amplifier (ISO-80; World Precision Instruments). The two needle electrodes were introduced subcutaneously into the root of the anterior limbs. The signal from the amplifier was monitored by a digital storage oscilloscope (TDS1000C-EDU; Tektronix, Tokyo, Japan) connected to a personal computer running the accessory software (OpenChoice; Tektronix, Tokyo, Japan) application software.

To evaluate the magnitude of signal *M* induced while varying FiO_2_, we calculated the change in signal based on time series data. The signal under normoxia was selected as a control *M*_c_, which was subtracted from each of the subsequent signals *M* in the series. Each subtracted value, which demonstrated the change in the signal, *M* − *M*_c_, over time, was normalized by dividing by *M*_c_. The change in signal is expressed as *∆M* = (*M* − *M*_c_)/*M*_c_. The above calculation was applied to the time series of *C*_HbO_, *C*_HbR_, *C*_HbT_, *StO*_2_, *b*, *SaO*_2_ and HR.

### 3.5. Statistical Considerations

A region of interest (ROI) was placed in each image for the analysis of time courses in *C*_HbO_, *C*_HbR_, *C*_HbT_, *StO*_2_, and *b*. Data are expressed as mean and standard deviation (SD). To compare whether the mean estimated value under each experimental condition differed significantly from the aggregate mean across experimental conditions, one-way repeated-measures analysis of variance (ANOVA) with Bonferroni’s correction was performed when comparing the relative change in signal among averages over the specific time periods. Bonferroni’s correction has more power detecting significant difference when the number of comparisons is small and is used to reduce the chances of obtaining false-positive results. Values of *p* < 0.05/*n* were considered statistically significant, where *n* is the number of samples. 

## 4. Conclusions

In summary, a method for imaging oxygenated hemoglobin concentration, deoxygenated hemoglobin concentration, total hemoglobin concentration, hemoglobin oxygen saturation, and the light scattering parameter of in vivo exposed brain tissues based on a single snap shot of an RGB image was demonstrated in the present study. In vivo experiments using exposed rat brain while changing the FiO_2_ confirmed the feasibility of the proposed method for visualizing both cortical hemodynamics and morphological changes due to loss of tissue viability in the brain. The results from this study indicate the potential of the method to evaluate pathophysiological conditions in brain tissues. The proposed method can capture both hemodynamic responses and morphological changes in brain tissue, and thus appears promising for monitoring brain function and tissue viability in neurosurgery as well as in the diagnosis of several neurological disorders, such as traumatic brain injury, seizure, ischemic stroke, and cerebral hyperperfusion syndrome. We intend to integrate the proposed method into a surgical microscope system to display brain tissue viability in real time on a monitor during neurosurgery. The proposed method has potential applicability in not only brain but also other organs such as the liver, heart, and kidney.
